# Supplementary material for: Splice-Junction-Based Mapping of Alternative Isoforms in the Human Proteome
Source: Cell Rep. Author manuscript; Available in PMC 2020 Jan 15. (PMC6961840; doi:10.1016/j.celrep.2019.11.026)
Supplement: 2 [file NIHMS1546469-supplement-2.zip › DF1/PXD010154/Heart/Heart_11_TTN_KPEAPPPKVPEVLPPK.pdf]

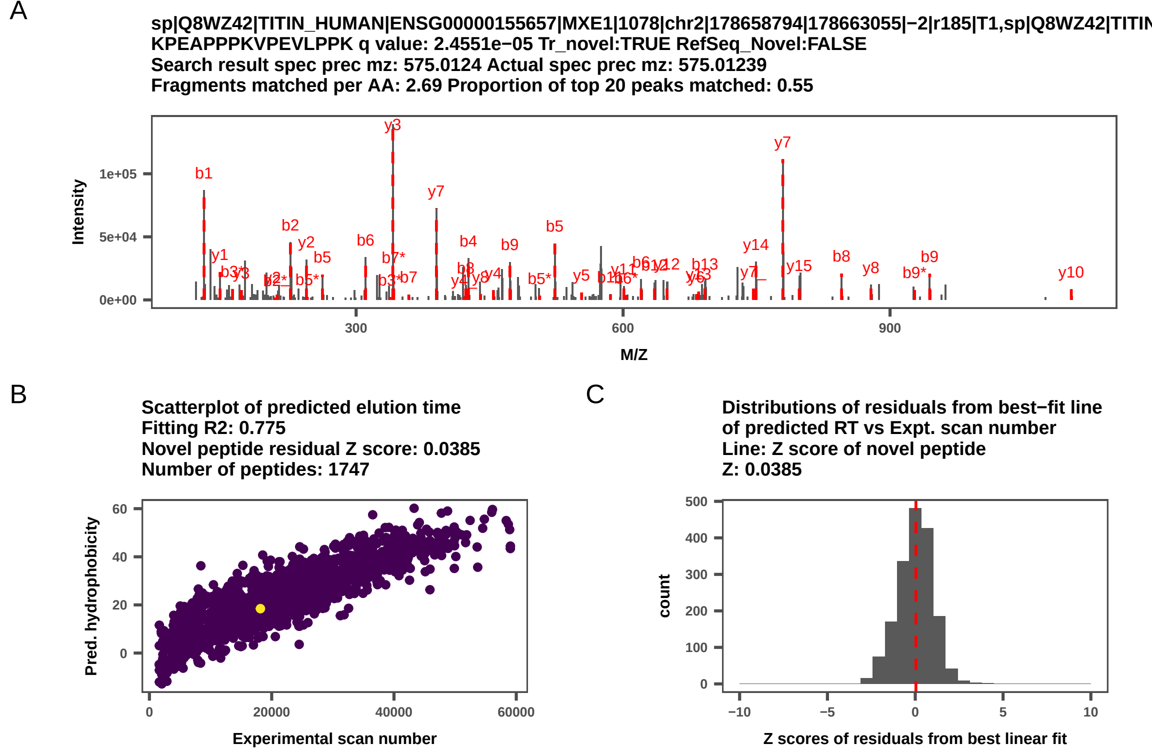
A
sp|Q8WZ42|TITIN_HUMAN|ENSG00000155657|MXE1|1078|chr2|178658794|178663055|−2|r185|T1,sp|Q8WZ42|TITIN
KPEAPPPKVPEVLPPK q value: 2.4551e−05 Tr_novel:TRUE RefSeq_Novel:FALSE
Search result spec prec mz: 575.0124 Actual spec prec mz: 575.01239
Fragments matched per AA: 2.69 Proportion of top 20 peaks matched: 0.55
Intensity
1e+05
5e+04
0e+00
300
600
900
M/Z
B
Scatterplot of predicted elution time
Fitting R2: 0.775
Novel peptide residual Z score: 0.0385
Number of peptides: 1747
Pred. hydrophobicity
60
40
20
0
0
20000
40000
60000
Experimental scan number
C
Distributions of residuals from best−fit line
of predicted RT vs Expt. scan number
Line: Z score of novel peptide
Z: 0.0385
count
500
400
300
200
100
0
−10
−5
0
5
10
Z scores of residuals from best linear fit
